# Cadaver Thanatomicrobiome Signatures: The Ubiquitous Nature of *Clostridium* Species in Human Decomposition

**DOI:** 10.3389/fmicb.2017.02096

**Published:** 2017-10-30

**Authors:** Gulnaz T. Javan, Sheree J. Finley, Tasia Smith, Joselyn Miller, Jeremy E. Wilkinson

**Affiliations:** ^1^Forensic Science Program, Physical Sciences Department, Alabama State University, Montgomery, AL, United States; ^2^Physical Sciences Department, Alabama State University, Montgomery, AL, United States; ^3^Research and Testing Laboratory, RTL Genomics, Lubbock, TX, United States

**Keywords:** thanatomicrobiome, *Clostridium*, 16S rRNA gene, V4 hypervariable regions, V3-4 hypervariable regions, Postmortem Clostridium Effect

## Abstract

Human thanatomicrobiome studies have established that an abundant number of putrefactive bacteria within internal organs of decaying bodies are obligate anaerobes, *Clostridium* spp. These microorganisms have been implicated as etiological agents in potentially life-threatening infections; notwithstanding, the scale and trajectory of these microbes after death have not been elucidated. We performed phylogenetic surveys of thanatomicrobiome signatures of cadavers’ internal organs to compare the microbial diversity between the 16S rRNA gene V4 hypervariable region and V3-4 conjoined regions from livers and spleens of 45 cadavers undergoing forensic microbiological studies. Phylogenetic analyses of 16S rRNA gene sequences revealed that the V4 region had a significantly higher mean Chao1 richness within the total microbiome data. Permutational multivariate analysis of variance statistical tests, based on unweighted UniFrac distances, demonstrated that taxa compositions were significantly different between V4 and V3-4 hypervariable regions (*p* < 0.001). Of note, we present the first study, using the largest cohort of criminal cases to date, that two hypervariable regions show discriminatory power for human postmortem microbial diversity. In conclusion, here we propose the impact of hypervariable region selection for the 16S rRNA gene in differentiating thanatomicrobiomic profiles to provide empirical data to explain a unique concept, the Postmortem Clostridium Effect.

## Introduction

Thanatomicrobiome studies have determined that there is extremely rapid postmortem overgrowth of *Clostridium* spp. within decaying internal body sites (e.g., blood, bone marrow, liver, prostate) (Clement et al., 2016; Javan et al., 2016a,b, Adserias-Garriga et al., 2017a,b; Thomas et al., 2017; Zhao et al., 2017). Human decomposition is a multifactorial process mediated by microbes, which inhabit, proliferate, and die externally and internally throughout dead biomass (Javan et al., 2016a,b). *Clostridium* spp. are strict anaerobes and common symbiotic bacteria located in healthy intestines. High abundance of nine *Clostridium* spp., namely *C. sordellii*, *C. difficile*, *C. bartlettii*, *C. bifermentans*, *C. limosum*, *C. haemolyticum*, *C. botulinum*, and *C. novyi*, were discovered by next-generation sequencing of 16S rRNA gene amplicons in previous thanatomicrobiome studies of human postmortem samples (Can et al., 2014; Javan et al., 2016b). Enteric bacteria, including *Clostridium* spp., are capable of translocating to surrounding tissues within 5–48 h after death at 25°C (Morris et al., 2006). *Clostridium* spp. reside in the mucosal layer and the intestinal epithelial monolayer, and they metabolize predigested hexoses entering from the stomach to acetic acid, acetone, butanoic acid, butanol, and ethanol which bacteria then ferment to pyruvate (Corry, 1978; Boumba et al., 2008; Janaway et al., 2009). Furthermore, Clostridia break down the amino acid threonine to propanol using threonine dehydratase, α-ketobutyrate synthase, and NAD-linked propanol dehydrogenase (Boumba et al., 2008).

Prokaryotic 16S rRNA gene amplicon sequences are extensively used in forensic microbiology as reliable biomarkers for taxonomic classification and phylogenetic analysis of the microbiome of death. The thanatomicrobiome, which is defined as microbial succession in decomposing remains (e.g., blood, bone marrow, liver, reproductive organs), can provide evidence concerning interactions between microorganisms and their mammalian hosts. Microbes symbiotically cohabitate with humans during life, but they also participate in the nature and trajectory of decomposition. The host’s death introduces chaos in microbial communities as the body becomes an abounding source of nutrients (Mondor et al., 2012). The question then arises, “What hypervariable region(s) of the 16S rRNA gene best profile the shifts that occur in response to the massive proliferation of microbiota after death?” The phenomenon that these signatures are left behind by the corpse provides unique forensic potential to make available trace evidence that can be used in microbial forensics.

Analysis of the very informative 16S rRNA gene is commonly used as a genetic marker for profiling prokaryotic communities (Lane et al., 1985; Woese et al., 1990; Baker et al., 2003; Tringe and Hugenholtz, 2008; Wang and Qian, 2009). Recent postmortem microbiome studies have focused on the 16S rRNA gene Class I, which spans the V4 region (Hyde et al., 2013, 2015; Damann et al., 2015; Javan et al., 2016b; Metcalf et al., 2016). The V4 hypervariable region is one of the major functional parts of the microbial gene because it encompasses a portion of the “690 hairpin” (Morosyuk et al., 2000; Wimberly et al., 2000) and decoding center (Schluenzen et al., 2000; Morosyuk et al., 2001). The V3 region is categorized in Class II, which is peripheral to the two functional centers of the 16S rRNA gene (Schluenzen et al., 2000; Schuwirth et al., 2005). Studies have shown that V4 is the best region for phylogenetic studies, particularly at the phylum level (Yang et al., 2016).

A key question is, which sub-region (V4 or V3-4) is more effective for phylogenetic studies of the human thanatomicrobiome? To explore the potential to determine cadaver thanatomicrobiome signatures using two hypervariable regions of the 16S rRNA gene, we compared the performance of primers 515F-806R (V4) to 357wF-785R (conjoined V3-4) hypervariable regions (Johnson et al., 2016). We hypothesized that by modulating 16S rRNA gene hypervariable fragment lengths on the Illumina MiSeq platform, the two specified regions would produce dissimilar microbial signatures.

Bioinformatic surveys have shown that hypervariable regions of 16S rRNA gene differ in the detection of sequence diversity; thus, a particular region may function well for ascertaining a spectrum of bacterial taxa whereas a different region may exhibit a distinct degree of taxonomic diversity. The V3-4 amplicon has a read length of twice 250 bp that offers an ideal target for Illumina paired-end sequencing and will provide a suitable framework for V4 region comparisons of the effectiveness of hypervariable region performance. Here, we performed a phylogenetic assessment of species distinctions using V4 versus V3-4 hypervariable regions from postmortem liver and spleen samples from criminal cases. Furthermore, we determined for the first time that fast-growing members of postmortem microbial communities, *Clostridium* spp., that usually predominate at longer PMIs, also are the most prominent prokaryotes even at shorter time intervals (PMI = 4 h).

## Materials and Methods

### Postmortem Sampling of Human Corpses

Postmortem samples included 28 male and 17 female corpses from the Alabama Department of Forensic Sciences in Montgomery, AL and The Office of the District One Medical Examiner in Pensacola, FL, United States. Demographic data were collected on each of the 45 corpses (i.e., age, gender, ethnicity, cause of death, PMI) (**Supplementary Table S1**). The study was approved by Alabama State University Institutional Review Board (IRB) number 2016011. Time of death of each corpse was certified from official Daily Crime Logs created by local police departments. Bodies were stored in the morgues at 1°C until time of tissue dissection. Approximately 10 mg of liver and spleen tissues were dissected using sterile scalpels and placed in polyethylene bags in an examination area at 20°C. Organs were transported on dry ice to the Thanatos Laboratory at Alabama State University. Specimens were stored at -80°C until time of DNA extraction.

### DNA Extraction of Postmortem Samples

Approximately 10 mg of thawed liver and spleen tissues were placed into Lysing matrix E tubes (MP Biomedicals) containing zirconia and silica beads, 0.5 ml phenol/chloroform/isoamyl alcohol (25:24:1) (TE saturated, pH 8.0) and 0.5 ml of 2× TENS buffer [100 mM Tris–HCl (pH 8.0), 40 mM EDTA, 200 mM NaCl, 2% SDS] (Wan et al., 2011). Tubes were homogenized by mechanical horizontal vortexing in a Mini Beadbeater (BioSpec Products) at speed 40 and time 6, briefly cooled on ice, and centrifuged at 16,000 rpm for 5 min. Supernatants were transferred to 2.0 ml Phase Lock Gel tubes (Invitrogen) containing 0.3 ml of 7.5 M ammonium acetate and equal volumes of chloroform. Tubes were mixed by repeated moderate inverting 10 times and supernatants were transferred into new tubes containing 0.6 volumes of ice cold isopropanol and 3 μl of GlycoBlue Coprecipitant (Life Technologies). After gently inverting several times, samples were incubated at -80°C for 10 min. Following centrifugation at 16,000 rpm for 5 min, isopropanol was decanted and pellets were washed with cold 80% ethanol and allowed to dry for 5 min. Pellets were eluted with 100 μl of TE buffer. DNA was quantified by NanoDrop2000^TM^ (Thermo Scientific) measuring the absorbance at 260 nm.

### Illumina MiSeq Sequencing

V4 and V3-4 hypervariable regions of 16S rRNA gene were amplified for sequencing at RTL Genomics (Research and Testing Laboratory, Lubbock, TX, United States) in two-step, independent reactions using HotStar Taq Master Mix Kit (Qiagen) with universal primers 515F-806R for the V4 region and primer constructs 357wF/785R for the longer, combined V3-4 regions. Primers for the first step were constructed using the fragment-specific forward and reverse primers (515F-806R or 357wF-785R) with the Illumina i5 and i7 sequencing primers added to the 5′-end of each, respectively. Products from the first amplification were added to a second PCR step based on qualitatively determined concentrations (amplicons were run on a 2% ethidium gel, gel bands were scored, and a volume of products was added to the second PCR based on the scores). Primers for the second PCR step were designed using Illumina Nextera PCR primers with 8 bp dual indexes. Each PCR amplification included 9 μl of sterile deionized H_2_O, 0.5 μl of 5 μM forward primer, 0.5 μl of 5 mM reverse primer, 1 μl of DNA template, and 14 μl of Taq Master Mix. The negative control was a reaction mixture with no template DNA. PCR reaction conditions included initial denaturation at 95°C for 5 min, then 25 cycles of 94°C for 30 s, annealing at 54°C for 40 s, and extension at 72°C for 1 min, followed by 1 cycle of 72°C for 10 min and 4°C hold. Barcoding PCR reactions were conducted under the same conditions, except with only 10 cycle extensions. Amplification products were visualized with eGels (Life Technologies). Products were then pooled equimolar and each pool was size selected in two rounds using SPRIselect beads (BeckmanCoulter) in a 0.7 ratio for both rounds. Size selected pools were then quantified using Qubit 2.0 fluorometer (Life Technologies) and loaded on an Illumina MiSeq 2x300 flow cell at 10 pM and sequenced.

### Bioinformatic Analysis

The sequence data were analyzed using a standard microbial diversity analysis pipeline, which consisted of two major stages, denoising and chimera detection followed by microbial diversity analysis. Denoising was performed using various techniques to remove short sequences, singleton sequences, and noisy reads. Chimera detection was performed using the UCHIME chimera detection software in *de novo* mode (Edgar et al., 2011). Lastly, remaining sequences were then corrected base-by-base to help remove noise from within each sequence. During the diversity analysis stage, each sample was run through the analysis pipeline to cluster reads into OTUs (at 97% identity) using the UPARSE algorithm (Edgar, 2013), and then globally aligned using the USEARCH global algorithm (Edgar, 2010) against a database of high-quality 16S rRNA gene sequences to determine taxonomic classifications. After OTU selection was performed, a phylogenetic tree was constructed in Newick format from a multiple sequence alignment of OTUs done in MUSCLE (Edgar, 2004a,b) and generated in FastTree (Price et al., 2009, 2010; Shah et al., 2016).

Microbial diversity of cadaver samples was examined from two perspectives using the phyloseq package in R (McMurdie and Holmes, 2013). First, overall richness (i.e., number of distinct nucleic acid sequences present within the microbiome) was expressed as the number of OTUs and was quantified using the Chao1 richness estimator. Secondly, overall microbial diversity, determined by both richness and evenness and the distribution of abundance among distinct taxa, was expressed as Shannon–Wiener species diversity. Measures of microbial diversity were screened for group (region, organ, gender, manner of death, PMI, season, location, weight, and height) differences using an analysis of variance (ANOVA). Multivariate differences among groups were evaluated with permutational multivariate analysis of variance (PERMANOVA) using distance matrices function ADONIS (Oksanen, 2011). For PERMANOVA, ADONIS distances among samples first were calculated using unweighted or weighted UniFrac via the phyloseq package in R (McMurdie and Holmes, 2013), and then an ANOVA-like simulation was conducted to test for group differences. Principal coordinates analysis (PCoA) using unweighted and weighted UniFrac distances and relative abundance bar plots were generated to visualize relationships and differences among groups. All analyses were conducted in R (R Development Core Team, 2010) and all plots were generated using the ggplot2 package (Wickham, 2009).

## Results

### Thanatomicrobiome Sequencing of Postmortem Liver and Spleen Samples

**FIGURE 1:**
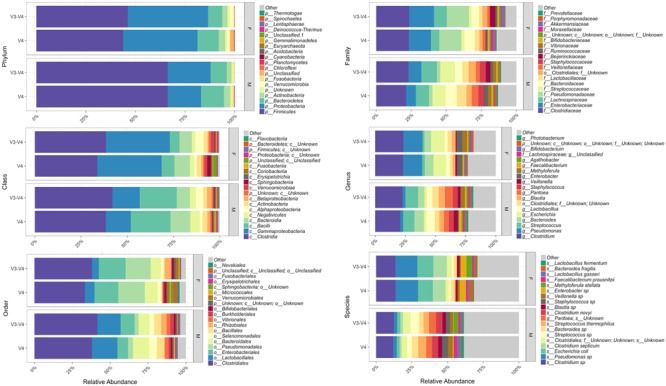
Relative abundances of the top most abundant bacteria in both 16S rRNA gene hypervariable region groups. Both region groups are illustrated according to taxonomic hierarchy and are faceted by gender.

Bioinformatic characterization of relative abundances and microbial diversity of the thanatomicrobiome was performed through metagenomic analyses in order to determine if there was greater discriminatory power exhibited by 16S rRNA gene V4 versus V3-4 hypervariable region amplicons. Operational taxonomic unit (OTU) data were validated by rarefaction analyses. Rarefaction data confirmed complete coverage until 20,000 sequences to observe all taxa as shown by convergence of vertical asymptotes for all curves (data not shown). Relative abundances of the top most abundant bacteria according to taxonomic hierarchy are shown in **Figure 1**. The highest percentage of bacteria on the order level was Clostridiales and seven of the top species were *Clostridium* spp. Furthermore, 95% of samples contained *Clostridium* spp., whereas six of the seven samples that did not contain *Clostridium* spp. were from the V3-4 hypervariable region.

### Thanatomicrobiome Alpha and Beta Diversity Analyses

**FIGURE 2:**
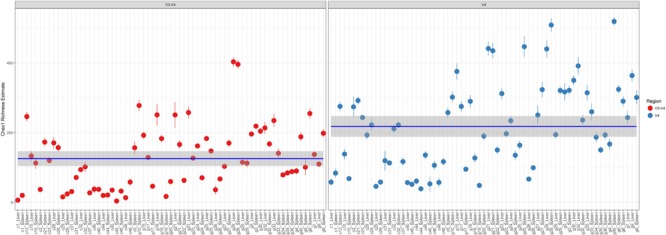
Chao1 richness within the total microbiome data. Richness estimates are colored (red dots, V3-4 regions and blue dots, V4 region) and faceted by 16S rRNA gene hypervariable region group. Mean Chao1 richness estimates (and confidence intervals) for each region group are also illustrated.

Comparison of Chao1 richness estimations was calculated and the V4 hypervariable region had a higher proportion of average calculated estimates than V3-4 (**Figure 2**). For V4 region amplicons, the average Chao1 richness estimate was 217 species, whereas V3-4 averaged 125 species. Also, ANOVA analysis revealed a statistically significant difference in Chao1 richness between two 16S rRNA gene regions (ANOVA; *p* < 0.001) (**Table 1**). In comparisons of gender and manner of death (accident, homicide, natural, suicide, undetermined), statistically significant differences were observed; however, patterns of species richness were statistically independent of time of death. Conversely, no significance was observed in Chao1 richness in the comparison of other variables (e.g., season of death).

**Table 1:** Results of ANOVA, that tested for differences in Chao1 richness.

	*df*	Sum Sq.	Mean Sq.	*F*-value	Pr (>*F*)
Region	1	294,432.16	294,432.16	53.02	0
Organ	1	9994.93	9994.93	1.8	1.822
Gender	1	109,506.49	109,506.49	19.72	0
Manner of death	4	555,725.31	138,931.33	25.02	0
PMI	1	21.15	21.15	0	0.9509
Season	3	73,761.48	24,587.16	4.43	0.0054
Weight	1	16.79	16.79	0	0.9562
Height	1	48,683.31	48,683.31	8.77	0.0037
Residuals	123	682,989.39	5552.76		

*Degrees of freedom (df) corresponds to one less than the number of values in the set of means. The *p*-values are derived from the *F*-distribution and the significance level is Pr (>*F*) < 0.001 (shaded cells)*.

**FIGURE 3:**
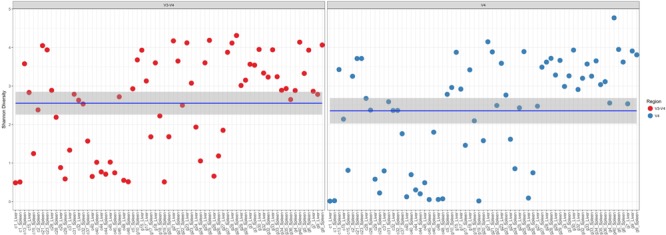
Shannon diversity within the total microbiome data. Diversity estimates are colored (red dots, V3-4 regions and blue dots, V4 region) and faceted by 16S rRNA gene hypervariable region group. Mean values (and confidence intervals) in each group are also illustrated. The mean Shannon diversity for both groups is approximately 2.5.

In a comparison of the Shannon Diversity within the total microbiome data, both hypervariable regions demonstrated an overall similar profile (**Figure 3**). The average Shannon Diversity index representing both regions was approximately 2.63 for the V4 region and 2.55 for V3-4; however, significant differences were observed for gender, manner of death, and season of death (ANOVA; *p* < 0.001, **Table 2**).

**Table 2:** Results of ANOVA, that tested for differences in Shannon diversity.

	*df*	Sum Sq.	Mean Sq.	*F*-value	Pr (>*F*)
Region	1	1.34	1.34	1.69	0.196
Organ	1	0.29	0.29	0.37	0.5467
Gender	1	13.67	13.67	17.21	0.0001
Manner of death	4	62.8	15.7	19.76	0
PMI	1	3.98	3.98	5.01	0.027
Season	3	15.73	5.24	6.6	0.0004
Weight	1	0.2	0.2	0.25	0.6153
Height	1	8.56	8.56	10.78	0.0013
Residuals	123	97.74	0.79		

*Degrees of freedom (df) corresponds to one less than the number of values in the set of means. The *p*-values are derived from the *F*-distribution and the significance level is Pr (>*F*) < 0.001 (shaded cells)*.

Results of multivariate difference interactions between both 16S rRNA gene regions and other variables resulted in statistical significance in region and location (interaction ADONIS; *p* = 0.001), which demonstrated that location was the only factor that had a confounding effect on region and that other factors were not confounding results. Results of ADONIS based on weighted UniFrac distances demonstrated significant differences among gender, manner of death, and season (*p* < 0.001), but not between V4 and V3-4 16S rRNA gene regions (**Table 3**). Given the fact that significant differences were observed among regions in unweighted but not in weighted UniFrac, the presence or absence of OTUs was more dissimilar than the abundance of OTUs (**Table 3**).

**Table 3:** Results of the permutational multivariate analysis of variance using distance matrices function ADONIS, unweighted and weighted Unifrac, respectively.

	Unweighted UniFrac						Weighted UniFrac					
	
	*df*	Sum Sq.	Mean Sq.	*F*-model	*R*^2^	Pr (>*F*)	*df*	Sum Sq.	Mean Sq.	*F*-model	*R*^2^	Pr (>*F*)
Region	1	1.5	1.5	8.35	0.05	0.001	1	0.15	0.15	1.26	0.01	0.26
Organ	1	0.19	0.19	1.06	0.01	0.284	1	0.08	0.08	0.65	0	0.644
Gender	1	0.49	0.49	2.72	0.02	0.002	1	0.91	0.91	7.71	0.04	0.001
Manner of death	4	2.52	2.52	3.51	0.08	0.001	4	2.04	0.51	4.29	0.09	0.001
PMI	1	0.33	0.33	1.85	0.01	0.025	1	0.62	0.62	5.19	0.03	0.002
Season	3	1.3	0.43	2.41	0.04	0.001	3	1.61	0.54	4.52	0.07	0.001
Residuals	123	22.07	0.18		0.71		123	14.6	0.12		0.67	
Total	137	29.78			1		137	21.87			1	

*Degrees of freedom (df) corresponds to one less than the number of values in the set of means. The *p*-values are derived from the *F*-distribution and the significance level is Pr (>*F*) < 0.001 (shaded cells)*.

**FIGURE 4:**
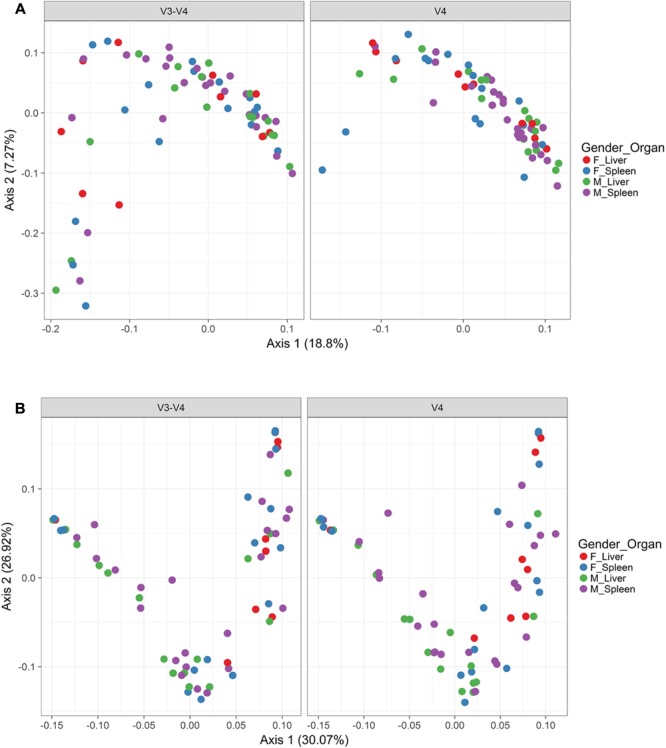
Principle coordinates analysis (PCoA) based on Unifrac distances. **(A)** PCoA based on unweighted Unifrac distances colored by gender and organ (red dots, female livers; blue dots, female spleens; green dots, male livers; purple dots, male spleens) and faceted by 16S rRNA gene hypervariable region. **(B)** PCoA based on weighted Unifrac distances colored by gender and organ (red dots, female livers; blue dots, female spleens; green dots, male livers; purple dots, male spleens) and faceted by 16S rRNA gene hypervariable region.

**FIGURE 5:**
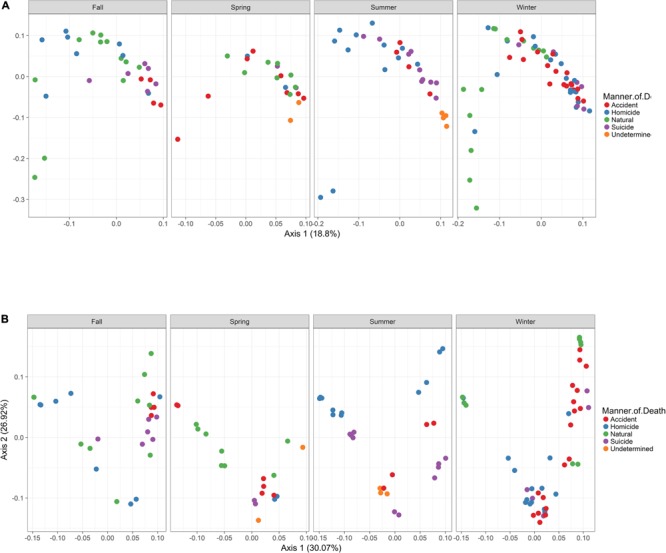
Principle Coordinates Analysis (PCoA) based on Unifrac distances. **(A)** PCoA based on unweighted Unifrac distances colored by manner and season (red dots, accident; blue dots, homicide; green dots, natural; purple dots, suicide; orange dots, undetermined). **(B)** PCoA based on weighted Unifrac distances colored by manner and season (red dots, accident; blue dots, homicide; green dots, natural; purple dots, suicide; orange dots, undetermined).

In order to visualize beta diversity differences between 16S rRNA gene regions, PCoA plots were generated based on unweighted (**Figure 4A**) and weighted Unifrac distances metrics (**Figure 4B**). For unweighted UniFrac, there was relatively low variance among two 16S rRNA gene regions, with only 18.10% of variance explained by primary Axis 1 and 7.27% explained by secondary Axis 2. PCoA plots based on unweighted and weighted Unifrac distances were generated faceted by manner of death and season (**Figures 5A,B**, respectively). Furthermore, samples for the V4 region clustered compactly together more than those for V3-4. For weighted UniFrac, there was more variance among 16S rRNA gene regions compared to unweighted UniFrac PCoA, with 30.07% of the variance explained by primary Axis 1 and 26.92% explained by secondary Axis 2.

## Discussion

Human antemortem microbiotas are well documented by the Human Microbiome Project (Peterson et al., 2009), for various body locations of healthy individuals; however, currently there is a paucity of knowledge and need for an in-depth interpretation concerning postmortem microbial communities of internal body sites. Our thanatomicrobiome research represents the largest exploratory study examining a cohort of 45 corpses in fresh and bloat stages. Also, the study provides the first extensive catalog of postmortem microbiomes obtained in internal locations analyzed by two different hypervariable regions of the 16S rRNA gene. Approximately 95% of the postmortem liver and spleen profiled in this study involved *Clostridium* spp. Moreover, the findings revealed that V4 and V3-4 hypervariable testings represent incongruent phylotype diversity and consequently support individual representative assessments of the thanatomicrobiome. For example, study-specific disparities were observed; *Clostridium* spp. were not obtained in only one of the V4 region sequences. On the other hand, these species were not obtained in six V3-4 region analyses. Here, we demonstrate that amplicons more sufficient to discriminate *Clostridium* spp. in postmortem tissue are derived from the V4 hypervariable region. According to previous thanatomicrobiome studies, *Clostridium* spp. predominated at long PMIs (up to 10 days) (Javan et al., 2016b). However, the current study determined that these Gram-positive, anaerobic extremophiles also predominate at shorter PMIs (4 h).

Our results support Yang et al. (2016) study based on geodesic distances that suggested V4 was the best sub-region for phylogenetic analysis. In the present study, we confirmed that the V4 region, belonging to Class I, had elevated sensitivity for the detection of forensically relevant bacteria; whereas V3 from Class II showed moderate sensitivity. Of particular interest, there was a higher enrichment of the species *Methyloferula stellata* discovered by targeting V3-4 amplicons compared to V4 (**Figure 1**). *M. stellata* is a methanotroph that grows exclusively on methane and methanol (Gill and Landi, 2011; Vorobev et al., 2011). During the bloat stage of human decomposition, methane, along with various odoriferous putrefaction gases, is produced in high abundance by anaerobic fermentation especially emanating from the gastrointestinal tract (Gill and Landi, 2011). Our study is the first to confirm a bacterial taxon that thrives on one of the putrefying gasses produced during decomposition through the use of 16S rRNA gene V3-4 combined regions in human internal body sites. Another very interesting finding was high abundances of three bacteria, *Escherichia coli*, *C. septicum*, and a *Pseudomonas* sp. detected only in female cases using both hypervariable regions (**Figure 1**).

In the last decade, postmortem microbiology studies have created novel thanatomicrobiome and epinecrotic communities catalogs using expertise in genetics, next-generation sequencing, and bioinformatics. The creation of a Human Postmortem Microbiome Project (HPMP) will facilitate the development of *modus operandi* used to empower data comparisons obtained from different national and international laboratories. Extension of existing standard operating procedures that cover sampling, processing, sequencing, and analysis will conceive universal standards for microbial analysis to unify the global research community. In addition, the HPMP framework includes research emphases that will provide the scientific community with a hub through which researchers can explore microbial life after death.

### Postmortem Clostridium Effect

The current research defines a new scientific concept, the “Postmortem Clostridium Effect” (PCE), which refers to facultative anaerobic *Clostridium* spp. that are ubiquitous during human decomposition. There are three dynamics that contribute to *Clostridium* species’ omnipresence in decaying humans; one factor involves its very fast doubling time. For example, a species found in the present study, *C. perfringens*, has the most rapid generation time of approximately 7.4 min at optimal temperatures (37–45°C) (Willardsen et al., 1979). The second factor is the bacterium’s proteolytic functions. *Clostridium* spp. have collagenases that digest native vertebrate collagen fibers which confer the ability to breach colon epithelial surfaces and mucosal layers and transmigrate to proximate tissues (Harrington, 1996; Burcham et al., 2016). The last advantageous putrefactive factor of *Clostridium* spp. develops via the cessation of the human heart which results in hypoxia (Gevers, 1975; Proskuryakov et al., 2003). A corpse that lacks oxygenated blood can facilitate enteric anaerobic bacteria, naturally found in the colon (e.g., *Clostridium* spp.), to efficiently and rapidly flourish in the nutrient-rich host. Previous human decomposition studies reported a marked shift from communities dominated by aerobic bacteria to anaerobic at the end of the bloat stage (Hyde et al., 2013). Taken together, these host–microbe factors within decaying biomass lead to the efficient functioning of the PCE.

## Conclusion

The thanatomicrobiome contributes a substantial function in modulating human decomposition. Studies are needed to elucidate if hypervariable regions are capable to discriminate all bacterial species; therefore, our emphasis was on the phylogenic resolution produced by small subunit surveys to characterize microbial mediators of decay. Conceivably, thanatomicrobiome analysis will be used to build predictive Thanatos models employing the PCE that can further designate the recovery of distinct community types associated with postmortem microbial communities.

## Ethics Statement

The study was approved by the Committee for the Protection of Human Subjects, Alabama State University Institutional Review Board (IRB) number 2016011. Methods were in accordance with relevant guidelines and regulations regarding working with cadavers. Written informed consent was obtained from next-of-kin relatives of the cases.

## Availability of Data and Material

All data generated or analyzed during this study are included in this published article and its supplementary information files.

## Author Contributions

GJ designed the study and collected human corpses. GJ, SF, JM, and TS extracted genomic DNA, PCR, gel electrophoresis the samples. JW performed MiSeq sequencing and data analysis. GJ and SF wrote and edited the article. All authors read and approved the final manuscript.

## Conflict of Interest Statement

The authors declare that the research was conducted in the absence of any commercial or financial relationships that could be construed as a potential conflict of interest.

## Supplementary Material

TABLE S1 Metadata of 16S rRNA regions, age, gender, ethnicity, season, location, cause of death, PMI.

## References

[B1] Adserias-Garriga J. Hernandez M. Quijada N. M. Lázaro D. R. Steadman D. Garcia-Gil J. (2017a). Daily thanatomicrobiome changes in soil as an approach of postmortem interval estimation: an ecological perspective. *Forensic Sci. Int.* 278 388–395. 10.1016/j.forsciint.2017.07.017 28818754

[B2] Adserias-Garriga J. Quijada N. M. Hernandez M. Lázaro D. R. Steadman D. Garcia-Gil J. (2017b). Dynamics of the oral microbiota as a tool to estimate time since death. *Mol. Oral Microbiol.* 10.1111/omi.12191 [Epub ahead of print]. 28654195

[B3] Baker G. C. Smith J. J. Cowan D. A. (2003). Review and re-analysis of domain-specific 16S primers. *J. Microbiol. Methods* 55 541–555. 10.1016/j.mimet.2003.08.009 14607398

[B4] Boumba V. A. Ziavrou K. S. Vougiouklakis T. (2008). Biochemical pathways generating post-mortem volatile compounds co-detected during forensic ethanol analyses. *Forensic. Sci. Int.* 174 133–151. 10.1016/j.forsciint.2007.03.018 17452087

[B5] Burcham Z. M. Hood J. A. Pechal J. L. Krausz K. L. Bose J. L. Schmidt C. J. (2016). Fluorescently labeled bacteria provide insight on post-mortem microbial transmigration. *Forensic Sci. Int.* 264 63–69. 10.1016/j.forsciint.2016.03.019 27032615

[B6] Can I. Javan G. T. Pozhitkov A. E. Noble P. A. (2014). Distinctive thanatomicrobiome signatures found in the blood and internal organs of humans. *J. Microbiol. Methods* 106 1–7. 10.1016/j.mimet.2014.07.026 25091187

[B7] Clement C. Hill J. M. Dua P. Culicchia F. Lukiw W. J. (2016). Analysis of RNA from Alzheimer’s disease post-mortem brain tissues. *Mol. Neurobiol.* 53 1322–1328. 10.1007/s12035-015-9105-6 25631714 PMC5450164

[B8] Corry J. E. L. (1978). Possible sources of ethanol ante-mortem and postmortem-its relationship to biochemistry and microbiology of decomposition. *J. Appl. Bacteriol.* 44 1–56. 10.1111/j.1365-2672.1978.tb00776.x 344299

[B9] Damann F. E. Williams D. E. Layton A. C. (2015). Potential use of bacterial community succession in decaying human bone for estimating postmortem interval. *J. Forensic Sci.* 60 844–850. 10.1111/1556-4029.12744 25808627

[B10] Edgar R. C. (2004a). MUSCLE: multiple sequence alignment with high accuracy and high throughput. *Nucleic Acids Res.* 32 1792–1797. 15034147 10.1093/nar/gkh340 PMC390337

[B11] Edgar R. C. (2004b). “MUSCLE: low-complexity multiple sequence alignment with T-coffee accuracy,” in *Proceedings of the Computational Systems Bioinformatics Conference, 2004: ISMB/ECCB*, Stanford, CA.

[B12] Edgar R. C. (2010). Search and clustering orders of magnitude faster than BLAST. *Bioinformatics* 26 2460–2461. 10.1093/bioinformatics/btq461 20709691

[B13] Edgar R. C. (2013). UPARSE: highly accurate OTU sequences from microbial amplicon reads. *Nat. Methods* 10 996–998. 10.1038/nmeth.2604 23955772

[B14] Edgar R. C. Haas B. J. Clemente J. C. Quince C. Knight R. (2011). UCHIME improves sensitivity and speed of chimera detection. *Bioinformatics* 27 2194–2200. 10.1093/bioinformatics/btr381 21700674 PMC3150044

[B15] Gevers W. (1975). Biochemical aspects of cell death. *Forensic Sci.* 6 25–29. 10.1016/0300-9432(75)90220-4 765245

[B16] Gill J. R. Landi K. (2011). Putrefactive rigor: apparent rigor mortis due to gas distension. *Am. J. Forensic Med. Pathol.* 32 242–244. 10.1097/PAF.0b013e3181dd17b9 20375836

[B17] Harrington D. J. (1996). Bacterial collagenases and collagen-degrading enzymes and their potential role in human disease. *Infect. Immun.* 64 1885–1891. 8675283 10.1128/iai.64.6.1885-1891.1996 PMC174012

[B18] Hyde E. R. Haarmann D. P. Lynne A. M. Bucheli S. R. Petrosino J. F. (2013). The living dead: bacterial community structure of a cadaver at the onset and end of the bloat stage of decomposition. *PLOS ONE* 8:e77733. 10.1371/journal.pone.0077733 24204941 PMC3813760

[B19] Hyde E. R. Haarmann D. P. Petrosino J. F. Lynne A. M. Bucheli S. R. (2015). Initial insights into bacterial succession during human decomposition. *Int. J. Legal Med.* 129 661–671. 10.1007/s00414-014-1128-4 25431049

[B20] Janaway R. Percival S. Wilson A. (2009). “Decomposition of human remains,” in *Microbiology and Aging*, ed. Percival S. (New York City, NY: Humana Press), 313–334.

[B21] Javan G. T. Finley S. J. Abidin Z. Mulle J. G. (2016a). The thanatomicrobiome: a missing piece of the microbial puzzle of death. *Front. Microbiol.* 7:225. 10.3389/fmicb.2016.00225 26941736 PMC4764706

[B22] Javan G. T. Finley S. J. Can I. Wilkinson J. E. Hanson J. D. Tarone A. M. (2016b). Human thanatomicrobiome succession and time since death. *Sci. Rep.* 6:29598. 10.1038/srep29598 27412051 PMC4944132

[B23] Johnson H. R. Trinidad D. D. Guzman S. Khan Z. Parziale J. V. DeBruyn J. M. (2016). A machine learning approach for using the postmortem skin microbiome to estimate the postmortem interval. *PLOS ONE* 11:e0167370. 10.1371/journal.pone.0167370 28005908 PMC5179130

[B24] Lane D. J. Pace B. Olsen G. J. Stahl D. A. Sogin M. L. Pace N. R. (1985). Rapid determination of 16S ribosomal RNA sequences for phylogenetic analyses. *Proc. Natl. Acad. Sci. U.S.A.* 82 6955–6959. 10.1073/pnas.82.20.6955 2413450 PMC391288

[B25] McMurdie P. J. Holmes S. (2013). phyloseq: an R package for reproducible interactive analysis and graphics of microbiome census data. *PLOS ONE* 8:e61217. 10.1371/journal.pone.0061217 23630581 PMC3632530

[B26] Metcalf J. L. Xu Z. Z. Weiss S. Lax S. Van Treuren W. Hyde E. R. (2016). Microbial community assembly and metabolic function during mammalian corpse decomposition. *Science* 351 158–162. 10.1126/science.aad2646 26657285

[B27] Mondor E. B. Tremblay M. N. Tomberlin J. K. Benbow E. M. Tarone A. M. Crippen T. L. (2012). The ecology of carrion decomposition. *Nat. Educ. Knowledge* 3 21.

[B28] Morosyuk S. V. Lee K. SantaLucia J. Cunningham P. R. (2000). Structure and function of the conserved 690 hairpin in *Escherichia coli* 16 S ribosomal RNA: analysis of the stem nucleotides. *J. Mol. Biol.* 300 113–126. 10.1006/jmbi.2000.3852 10864503

[B29] Morosyuk S. V. SantaLucia J. Cunningham P. R. (2001). Structure and function of the conserved 690 hairpin in *Escherichia coli* 16 S ribosomal RNA. III. Functional analysis of the 690 loop. *J. Mol. Biol.* 307 213–228. 10.1006/jmbi.2000.4432 11243815

[B30] Morris J. A. Harrison L. M. Partridge S. M. (2006). Postmortem bacteriology: a re-evaluation. *J. Clin. Pathol.* 59 1–9. 10.1136/jcp.2005.028183 16394274 PMC1860254

[B31] Oksanen J. (2011). *Multivariate Analysis of Ecological Communities in R: Vegan Tutorial. R Package Version*. Available at: https://cran.r-project.org

[B32] Peterson J. Garges S. Giovanni M. McInnes P. Wang L. Schloss J. A. (2009). The NIH human microbiome project. *Genome Res.* 19 2317–2323. 10.1101/gr.096651.109 19819907 PMC2792171

[B33] Price M. N. Dehal P. S. Arkin A. P. (2009). FastTree: computing large minimum evolution trees with profiles instead of a distance matrix. *Mol. Biol. Evol.* 26 1641–1650. 10.1093/molbev/msp077 19377059 PMC2693737

[B34] Price M. N. Dehal P. S. Arkin A. P. (2010). FastTree 2–approximately maximum-likelihood trees for large alignments. *PLOS ONE* 2015:e9490. 10.1371/journal.pone.0009490 20224823 PMC2835736

[B35] Proskuryakov S. Y. Konoplyannikov A. G. Gabai V. L. (2003). Necrosis: a specific form of programmed cell death? *Exp. Cell Res.* 283 1–16. 10.1016/S0014-4827(02)00027-7 12565815

[B36] R Development Core Team (2010). *R: A Language and Environment for Statistical Computing*. Vienna: R Foundation for Statistical Computing.

[B37] Schluenzen F. Tocilj A. Zarivach R. Harms J. Gluehmann M. Janell D. (2000). Structure of functionally activated small ribosomal subunit at 3.3 Å resolution. *Cell* 102 615–623. 10.1016/S0092-8674(00)00084-2 11007480

[B38] Schuwirth B. S. Borovinskaya M. A. Hau C. W. Zhang W. Vila-Sanjurjo A. Holton J. M. (2005). Structures of the bacterial ribosome at 3.5 Å resolution. *Science* 310 827–834. 10.1126/science.1117230 16272117

[B39] Shah V. Luxton T. P. Walker V. K. Brumfield T. Yost J. Shah S. (2016). Fate and impact of zero-valent copper nanoparticles on geographically-distinct soils. *Sci. Total Environ.* 573 661–670. 10.1016/j.scitotenv.2016.08.114 27585433 PMC7384298

[B40] Thomas T. B. Finley S. J. Wilkinson J. E. Wescott D. J. Gorski A. Javan G. T. (2017). Postmortem microbial communities in burial soil layers of skeletonized humans. *J. Forensic Leg. Med.* 49 43–49. 10.1016/j.jflm.2017.05.009 28527363

[B41] Tringe S. G. Hugenholtz P. (2008). A renaissance for the pioneering 16S rRNA gene. *Curr. Opin. Microbiol.* 11 442–446. 10.1016/j.mib.2008.09.011 18817891

[B42] Vorobev A. V. Baani M. Doronina N. V. Brady A. L. Liesack W. Dunfield P. F. (2011). *Methyloferula stellata* gen. nov., sp. nov., an acidophilic, obligately methanotrophic bacterium that possesses only a soluble methane monooxygenase. *Int. J. Syst. Evol. Microbiol.* 61(Pt 10), 2456–2463. 10.1099/ijs.0.028118-0 21097638

[B43] Wan M. Rosenberg J. N. Faruq J. Betenbaugh M. J. Xia J. (2011). An improved colony PCR procedure for genetic screening of *Chlorella* and related microalgae. *Biotechnol. Lett.* 33 1615–1619. 10.1007/s10529-011-0596-6 21431847

[B44] Wang Y. Qian P. Y. (2009). Conservative fragments in bacterial 16S rRNA genes and primer design for 16S ribosomal DNA amplicons in metagenomic studies. *PLOS ONE* 4:e7401. 10.1371/journal.pone.0007401 19816594 PMC2754607

[B45] Wickham H. (2009). *ggplot2: Elegant Graphics for Data Analysis*. New York, NY: Springer-Verlag.

[B46] Willardsen R. R. Busta F. F. Allen C. E. (1979). Growth of *Clostridium perfringens* in three different beef media and fluid thioglycollate medium at static and constantly rising temperatures. *J. Food Prot.* 42 144–148. 10.4315/0362-028X-42.2.144 30812339

[B47] Wimberly B. T. Brodersen D. E. Clemons W. M. Jr. Morgan-Warren R. J. Carter A. P. Vonrhein C. (2000). Structure of the 30S ribosomal subunit. *Nature* 407 327–339. 10.1038/35030006 11014182

[B48] Woese C. R. Kandler O. Wheelis M. L. (1990). Towards a natural system of organisms: proposal for the domains Archaea, Bacteria, and Eucarya. *Proc. Natl. Acad. Sci. U.S.A.* 87 4576–4579. 10.1073/pnas.87.12.4576 2112744 PMC54159

[B49] Yang B. Wang Y. Qian P. Y. (2016). Sensitivity and correlation of hypervariable regions in 16S rRNA genes in phylogenetic analysis. *BMC Bioinformatics* 17:135. 10.1186/s12859-016-0992-y 27000765 PMC4802574

[B50] Zhao Y. Jaber V. Lukiw W. J. (2017). Secretory products of the human GI tract Microbiome and their potential impact on Alzheimer’s disease (AD): detection of lipopolysaccharide (LPS) in AD hippocampus. *Front. Cell. Infect. Microbiol.* 7:318. 10.3389/fcimb.2017.00318 28744452 PMC5504724

